# Targeting mitochondria to protect the heart: a matter of balance?

**DOI:** 10.1042/CS20258287

**Published:** 2025-12-08

**Authors:** Fouad A. Zouein, George W. Booz

**Affiliations:** 1 Department of Pharmacology and Toxicology, American University of Beirut Faculty of Medicine, Beirut, Lebanon; 2 Department of Pharmacology and Toxicology, School of Medicine, The University of Mississippi Medical Center, Jackson, MS, U.S.A.

**Keywords:** cardiac myocyte, ischemia-reperfusion injury, mitochondrial dynamics, mitochondrial fusion, myocardial infarction, mitochondrial fission

## Abstract

Mitochondria are dynamic, undergoing both fission and fusion. Evidence indicates that a balance between these two processes is necessary to maintain a healthy state. With ischemia/reperfusion (I/R) of the heart, fission is enhanced and is associated with mitochondrial swelling, depolarization, and production of reactive oxygen species, as well as apoptosis. Accumulating evidence indicates that blocking fission is effective in reducing I/R-induced tissue damage and contractile dysfunction. In theory, enhancing fusion should also serve to prevent I/R-related heart damage. In this perspective article, we present evidence from preclinical studies over the last several years supporting the conclusion that targeting mitochondrial dynamics is a promising pharmacological strategy to protect the heart. Such an approach has great value in limiting heart damage from not only myocardial infarction but also medical interventional reperfusion, alcohol consumption, chemotherapy, and sepsis.

## Introduction

**Figure 1:**
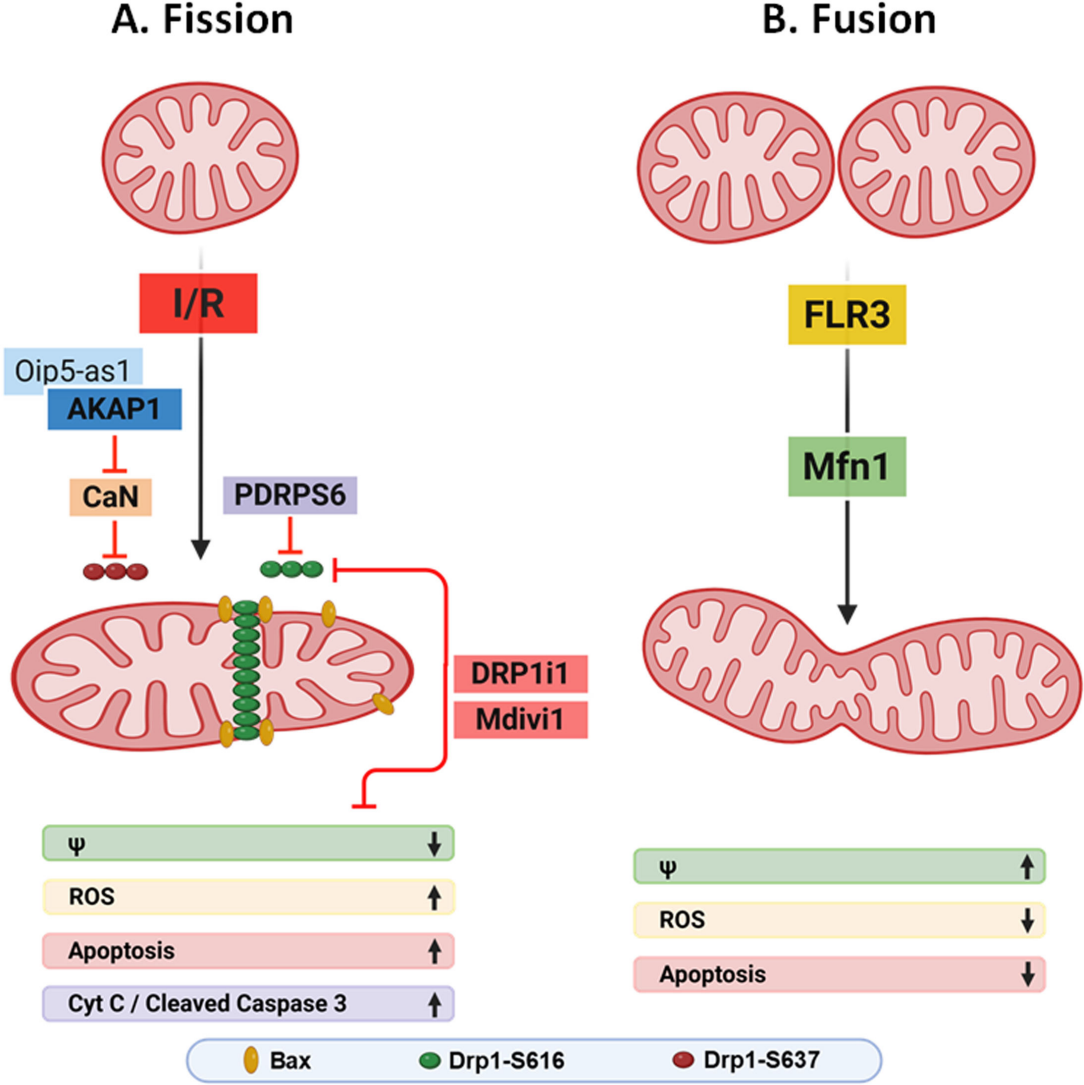
Mitochondrial dynamics and I/R injury. (**A**) With I/R, Drp1 translocates to mitochondria, where it oligomerizes and participates in the fission process. Fragmented mitochondria are more susceptible to swelling, loss of membrane potential (ψ), mitochondrial permeability transition pore (mPTP) opening, and ROS production (lower panel). Bax is recruited as well, enhancing mitochondrial outer membrane permeabilization, cytochrome C release, caspase activation, and apoptosis. Inhibiting Drp1 with mitochondrial division inhibitor 1 (mdivi-1) or DRP1i1 reduces I/R injury to the heart. The lncRNA, Oip5-as1, was recently shown to diminish mitochondrial fission by interacting with the scaffold protein A-kinase anchoring protein 1, AKAP1, thereby inhibiting calcineurin (CaN) phosphatase and maintaining Ser637 phosphorylation of Drp1, which inhibits its activity. Conversely, the peptide PDRPS6 interacts with phosphoglycerate mutase 5 (PGAM5), thereby inhibiting Drp1 activity by causing the dephosphorylation of Drp1 at Ser616. (**B**) The utility of the alternative approach of enhancing fusion was recently assessed in a study that used the synthetic flavagline, flavagline 3 (FL3), which was found to limit I/R injury and preserve cardiac function. FL3 promoted mitochondrial fusion by a mechanism dependent on Mfn1. See text for additional details. This presentation was created in BioRender. Drp1, dynamin-related protein 1; Mfn1, mitofusin 1.

Heart attacks are a leading cause of death worldwide with no effective pharmacological strategies to limit permanent damage to the heart. However, accumulating preclinical evidence has focused attention on targeting mitochondria, which occupy about a third of the volume of an adult cardiomyocyte and are a major source of damaging reactive oxygen species (ROS) with reperfusion. One approach involves tempering complex I activity so as to limit formation of ROS, for instance with metformin [1]. An alternative strategy is built around the fact that mitochondria are dynamic, undergoing fission or fusion, which is necessary for both the cell and mitochondria to maintain a healthy state. Normally, a balance occurs between fission and fusion, such that an increase in one over the other has harmful consequences. Indeed, in a seminal study, Song et al. [2] demonstrated that a lack of mitochondrial dynamism in cardiac myocytes is less deleterious than dynamic imbalance. Surprisingly, under nonstressed conditions, mitochondrial size (fragmentation or enlargement) would seem to be a less important determinant of cardiac phenotype or pathology than the impact that dynamic imbalance has via dysregulated mitochondria quality control, as evidenced by alterations in mitophagy or mitogenesis. The purpose of our article is to assess recent developments in studies that focused on targeting mitochondrial dynamics as a way to protect the heart from ischemia/reperfusion (I/R) injury, either by inhibiting fission or alternatively by enhancing fusion (Figure 1).

## Overview

The GTPase dynamin-related protein 1 (Drp1) plays a critical role in mitochondrial fission. In response to certain stimuli, cytosolic Drp1 translocates to mitochondria, where it oligomerizes at the future fission site and forms a ring. The GTP-dependent constriction of this ring leads to mitochondrial division. Several studies have documented increased mitochondrial fission in isolated cardiac myocytes of whole hearts with I/R [3–7]. Additionally, knockdown of Drp1 or its pharmacological blockade with mitochondrial division inhibitor 1 (mdivi-1) has been shown to have remarkable protective effects on cardiac myocyte viability and heart function. Besides the anticipated changes in mitochondrial size, diminishing the actions of Drp1 during I/R is associated with a reduction in infarct size, diastolic dysfunction, incidence of arrhythmias, ROS formation, cytochrome c release, caspase 3 activation, and apoptosis. Targeting Drp1 is most effective if done prior to ischemia but still effective if done during ischemia; however, conflicting results are reported for the effectiveness of targeting Drp1 during re-oxygenation [3,4]. Attenuating mitochondrial fission by targeting Drp1 may have relevance in sudden cardiac arrest as well [8]. Although not proven, the simplest explanation for the beneficial consequences of blocking fission is that, compared with elongated mitochondria, fragmented mitochondria are more susceptible to swelling and outer membrane permeabilization and less able to accommodate a calcium load and oxidative stress before undergoing mitochondrial permeability transition pore opening. Elongated mitochondria may have a higher respiratory capacity as well.

Recent findings support the conclusion that there are two distinct types of fission that are regulated by different molecular mechanisms [9]. Midzone division is associated with mitochondrial biogenesis and represents a physiological process. On the other hand, peripheral division is associated with the removal of damaged mitochondria, which are then targeted for degradation by autophagy or mitophagy. Peripheral division is generally accompanied by elevated concentrations of ROS. With midzone division, the outer mitochondrial membrane adaptor protein mitochondrial fission factor is involved in Drp1 recruitment, whereas the adaptor protein mitochondrial fission 1 protein is primarily responsible for recruiting Drp1 during peripheral division.

Peripheral fission accomplishment may also be dependent upon the final level of the pro-apoptotic protein Bax that is recruited to the mitochondrial outer membrane, which in turn may be determined by Drp1 [10]. During I/R, Bax translocates to the mitochondria and controls permeability of the mitochondrial outer membrane and cytochrome C release. In cardiac myocytes, DNA-dependent protein kinase catalytic subunit may promote I/R injury by suppressing Bax inhibitor-1 activity [11].

The role of mitochondrial fusion with I/R is unresolved, the key proteins being the outer membrane proteins and GTPases, mitofusin 1 and 2 (Mfn1 and Mfn2), and the inner membrane protein, optic atrophy 1. Reduced OPA1 protein levels are associated with ischemia [12], and Mfn2 activity is linked to suppression of Bax activation and free radical-induced mitochondrial depolarization [13]. But implicating these proteins in I/R injury is complicated by their extra-fusion functions; for instance, hearts deficient in both Mfn1 and Mfn2 are protected against acute infarction due to impaired mitochondria/SR tethering [14].

## Targeting I/R-induced mitochondrial fission

Ischemia-reperfusion injury is associated with enhanced fission and impaired mitophagy, leading to increased ROS-related cardiomyocyte damage. Lees et al. recently assessed the effectiveness of a novel small-molecule Drp1 inhibitor, Drp1 inhibitor 1, to protect against myocardial I/R injury in the mouse [15]. Using a cardiac-targeted nanoparticle drug delivery system, they observed significantly reduced infarct size and Ser616 phosphorylation of Drp1, a key regulatory mechanism for mitochondrial fission, and restored cardiomyocyte mitochondrial size to that of the sham group. Results from another study suggest that targeting the counteracting Ser637 phosphorylation site of Drp may also prove to be a viable way of limiting excessive fission in the I/R-injured heart [16]. Offsetting a decrease in the long non-coding RNA (lncRNA) Oip5-as1 diminished mitochondrial fission and myocardial infarct size and improved cardiac function in a mouse model of I/R injury. Evidence was found that Oip5-as1 selectively interacts with A-kinase anchoring protein 1, thereby inhibiting calcineurin (CaN) activation and subsequent Drp1 dephosphorylation at Ser637 [16]. Mitochondrial fission would be attenuated as translocation of Drp1 to the mitochondria is blocked.

Understanding how Drp1 phosphorylation is regulated may open new avenues for the pharmacological prevention of I/R injury. In that context, a recent study assessed the involvement of PDRPS6, a peptide derived from the 40S ribosomal protein S6, with hypoxic stress [17]. Delivery of PDRPS6, modified with a TAT sequence to enhance penetration, twenty minutes prior to reperfusion in rats, ameliorated myocardial tissue injury, cardiomyocyte apoptosis, and cardiac dysfunction. *In vitro* studies established that PDRPS6 decreased ROS levels and inhibited mitochondrial fission [17]. Evidence was provided that PDRPS6 leads to the dephosphorylation of Drp1 at Ser616 by interacting with the phosphoglycerate mutase 5 (PGAM5).

## Enhancing mitochondrial fusion to limit I/R injury

Cardioprotection may also result from manipulations that favor mitochondrial fusion, as evidenced by a recent study with the synthetic flavagline, flavagline 3 (FL3). Flavaglines are a family of natural products that have potent anticancer properties. Using a mouse model, FL3 was found to have major cardioprotective effects against myocardial I/R injury [18]. Pretreatment with FL3 reduced infarct size, improved cardiac function, and inhibited cardiomyocyte apoptosis. Additional posttreatment with FL3 attenuated chronic heart failure and cardiac fibrosis. Notably, FL3 promoted mitochondrial fusion by a mechanism dependent on Mfn1, which was necessary for its protective actions. These fusion-promoting effects of FL3 occur separately from its binding with prohibitins (PHBs) and their translocation to mitochondria and the inactivation of STAT3 signaling, which also contribute to cardioprotection. This study also showed that FL3 enhances mitochondrial-endoplasmic reticulum (ER) interactions, i.e. mitochondria-associated membranes, thereby helping to maintain calcium homeostasis and preventing mitochondrial calcium overload. The Mfn1-dependent actions of FL3 are independent of PHBs, specifically PHB1 and PHB2 [18]. These proteins play a crucial role in regulating mitochondrial fusion by acting as a scaffold on the inner mitochondrial membrane that regulates OPA1 activity, which is essential for inner membrane fusion and cristae maintenance.

However, as previously mentioned, the role of Mfn proteins in the heart is complex. For instance, evidence has been reported that Mfn2-deficient adult cardiomyocytes are protected from death-inducing stimuli, and Mfn2 knockout hearts recover better from reperfusion injury [19]. But there are separate ER and mitochondrial pools of MFn2 that are differentially regulated. Selective MARCH5-mediated ubiquitination of mitochondrial Mfn2 influences its activity and is important for tethering mitochondria to the ER [20]. However, with hypoxia, MARCH5-mediated ubiquitination promotes the degradation of mitochondrial Mfn2, resulting in reduced mitochondrial fusion and impaired mitochondrial function [21]. A recent *in vitro* study reported data supporting the feasibility of using a novel peptide, CVP-220, to specifically disrupt MARCH5-Mfn2 interactions and thereby protect cardiomyocytes from hypoxia-induced mitochondrial dysfunction [22]. Treatment with CVP-220 enhanced cell viability, reduced ROS formation, and selectively targeted MARCH5-mediated ubiquitination of Mfn2 without affecting other MARCH5 interactions. Thus, mitochondrial fusion was preserved, and mitochondrial fragmentation was prevented under stress conditions.

## Conclusion

Accumulating data support the conclusion that preserving mitochondrial homeostasis by targeting either fission or fusion is an effective strategy to protect the heart from I/R injury (Table 1). However, more needs to be known about how the processes of fission and fusion are regulated. Besides myocardial infarction and medical interventional reperfusion, targeting mitochondrial dynamics may have value in limiting heart damage from alcohol consumption [23], chemotherapy [24], obesity [25], and sepsis [26]. The finding that FL3 protects against cardiac I/R injury by enhancing mitochondrial fusion is especially exciting, as flavaglines have been shown to offer protection against doxorubicin-induced cardiotoxicity in preclinical studies, an adverse consequence of doxorubicin’s use in cancer chemotherapy. Overall, the results so far on fission and fusion have broad clinical implications and may herald the development of entirely new classes of cardioprotective drugs that target mitochondrial dynamics.

**Table 1:** Recent findings regarding the inhibition of fission and promotion of fusion in protecting the heart from I/R injury

Dynamic	Target	Preclinical findings I/R models
Fission	Drp1	Effective prior to and during ischemia; conflicting results of targeting during re-oxygenation
		Beneficial in the context of peripheral division
		Drp1 inhibitor 1 (DRP1i1) reduces Ser616 phosphorylation of Drp1 and infarct size
		lncRNA Oip5-as1 diminishes fission and myocardial infarct size, and improves cardiac function by inhibiting CaN-mediated Ser637 dephosphorylation of Drp1
		Peptide PDRPS6 ameliorates myocardial injury, cardiomyocyte apoptosis, and cardiac dysfunction by inhibiting fission via Ser616 dephosphorylation of Drp1
Fusion	Mfn1/Mfn2	Synthetic flavagline, FL3 promotes mitochondrial fusion by a mechanism dependent on mitofusin, Mfn1
		Novel peptide, CVP-220, disrupts MARCH5-mediated ubiquitination and degradation of mitochondrial Mfn2, which preserves fusion and protects cardiomyocytes from hypoxia-induced mitochondrial dysfunction

## Open Access

This article has been published open access under our Subscribe to Open programme, made possible through the support of our subscribing institutions, learn more here: https://portlandpress.com/pages/open_access_options_and_prices#conditional

## Abbreviations

AKAP1: A-kinase anchoring protein 1
CaN: calcineurin
Drp1: dynamin-related protein 1
ER: endoplasmic reticulum
FL3: flavagline 3
I/R: ischemia/reperfusion
MAM: mitochondria-associated membrane
Mfn1 and Mfn2: mitofusin 1 and 2
OPA1: optic atrophy 1
PHB: prohibitin
ROS: reactive oxygen species
lncRNA: long non-coding RNA
mdivi-1: mitochondrial division inhibitor 1
