# Correction: Targeting mitochondria to protect the heart: a matter of balance?

**DOI:** 10.1042/CS20258287_COR

**Published:** 2026-03-13

**Authors:** Fouad A. Zouein, George W. Booz

**Affiliations:** 1 Department of Pharmacology and Toxicology, American U­niversity of Beirut Faculty of Medicine, Beirut, Lebanon; 2 Department of Pharmacology and Toxicology, School of Medicine, The University of Mississippi Medical Center, Jackson, MS, U.S.A.

**Keywords:** cardiac myocyte, ischemia-reperfusion injury, mitochondrial dynamics, mitochondrial fusion, myocardial infarction, mitochondrial fission

The authors of the original article “Targeting mitochondria to protect the heart: a matter of balance?” (10.1042/CS20258287) would like to correct the Funding Statement. In the original article, the manuscript was inadvertently linked to the grant P20GM121334. The requested correction has been assessed and agreed to by the Editorial Board. The authors declare that these corrections do not change the results or conclusions of their paper.

The corrected version of Funding Statement is presented here:

“This work was supported by a grant to FAZ from the American University of Beirut Faculty of Medicine (MPP – 320145). GWB acknowledges the support of the Department of Pharmacology and Toxicology (UMMC)”.

